# Longitudinal analysis of humoral and cellular immunity in SARS-CoV-2 exposed families

**DOI:** 10.1038/s41598-025-07739-3

**Published:** 2025-07-18

**Authors:** Alex Dulovic, Armin Rabsteyn, Jonathan Remppis, Irene K. E. Gentzcke, Julia Mueller, Nadja Tuecks, Matthias Becker, Daniel Junker, Philipp D. Kaiser, Bjoern Traenkle, Ulrich Rothbauer, Juliane S. Walz, Andreas Peter, Sebastian Hörber, Tina Ganzenmueller, Thomas Iftner, Maximilian Stich, Burkhard Tönshoff, Philipp Henneke, Roland Elling, Klaus-Michael Debatin, Ales Janda, Nicole Schneiderhan-Marra, Axel R. Franz, Peter Lang, Hanna Renk

**Affiliations:** 1 NMI Natural and Medical Sciences Institute at the University of Tübingen, Reutlingen, Germany; 2 German Cancer Consortium (DKTK) and German Cancer Research Center (DKFZ), Partner Site Tübingen, Tübingen, Germany; 3 Cluster of Excellence iFIT (EXC2180) “Image-Guided and Functionally Instructed Tumor Therapies”, University of Tübingen, Tübingen, Germany; 4 Department of Pediatric Oncology and Hematology, University Children’s Hospital Tübingen, Tübingen, Germany; 5 Department of Neuropediatrics, General Pediatrics, Diabetology, Endocrinology and Social Pediatrics, University Children’s Hospital Tübingen, Tübingen, Germany; 6 Pharmaceutical Biotechnology, University of Tübingen, Tübingen, Germany; 7 Department of Peptide-based Immunotherapy, Institute of Immunology, University and University Hospital Tübingen, Tübingen, Germany; 8 Clinical Collaboration Unit Translational Immunology, Department of Internal Medicine, German Cancer Consortium (DKTK), University Hospital Tübingen, Tübingen, Germany; 9 Institute for Clinical Chemistry and Pathobiochemistry, University Hospital Tübingen, Tübingen, Germany; 10 Institute for Medical Virology and Epidemiology of Viral Diseases, University Hospital Tübingen, Tübingen, Germany; 11 Heidelberg University, Medical Faculty, Departement of Pediatrics I, , University Children‘s Hospital Heidelberg, Heidelberg, Germany; 12 Department of Infectious Diseases, Molecular Virology, University of Heidelberg, Heidelberg, Germany; 13 Division of Virus-Associated Carcinogenesis, German Cancer Research Center, Heidelberg, Germany; 14 German Center for Infection Research, Heidelberg Partner Site, Heidelberg, Germany; 15 Center for Pediatrics and Adolescent Medicine, Faculty of Medicine, Medical Center Freiburg, University of Freiburg, Freiburg, Germany; 16 Institute for Immunodeficiency, University Medical Center Freiburg, Freiburg, Germany; 17 Department of Pediatrics and Adolescent Medicine, Ulm University Medical Center, Ulm University, Ulm, Germany; 18 Center for Pediatric Clinical Studies, University Hospital Tübingen, Tübingen, Germany; 19 Department of Neonatology, University Children’s Hospital Tübingen, Tübingen, Germany

**Keywords:** Immunology, Medical research

## Abstract

Identification of previous SARS-CoV-2 infection typically relies on serology, yet T-cells play a key role in the adaptive immune response against SARS-CoV-2. Here, we investigated in parallel the SARS-CoV-2-specific as well as endemic human coronavirus-specific humoral and cross-reactive cellular responses in children and adults. We analyzed clinical data and blood samples from a family cohort of 96 children and 144 adults at 3–4 and 11–12 months after their first contact with SARS-CoV-2. Humoral response was assessed by a multiplex immunoassay with high sensitivity and specificity (MULTICOV-AB). Cellular responses were analyzed by IFN-γ ELISPOT using four different established epitope compositions (ECs) to discriminate between SARS-CoV-2 specific and HCoV cross-reactive T-cell responses. While the majority of adults had a combined serological and T-cell response, relatively more children had a T-cell response alone rather than a combined response. The magnitude of the T-cell response correlated with symptoms and the humoral response. In addition, SARS-CoV-2 infection significantly boosted the endemic coronavirus-specific cellular response. Overall, our data suggest discordant humoral and cellular responses, reflecting either abortive infection, cellular sensitization with rapid viral clearance or rapid antibody waning or a combination of these phenomena. Restricting epidemiologic analysis to SARS-CoV-2 serological data may underestimate rates of infection with or at least exposure to SARS-CoV-2 in children.

## Introduction

Early in the severe acute respiratory syndrome coronavirus 2 (SARS-CoV-2) pandemic, children appeared to be protected from severe disease. Lower hospitalization rates in children compared to adults reflect this finding^1,2^. This was still true as time went on and many different co-circulating variants due to random mutations of the SARS-CoV-2’s genome occurred. The milder clinical course of infection in children might not only be explained by differences in the kinetics of virus-specific antibodies and several innate immune factors, but also by differences in the adaptive cellular immunity between children and adults^3,4^. T-cells play an important role in the successful immune response to SARS-CoV-2 infection^5^. Early induction of SARS-CoV-2 specific T-cells was observed in a longitudinal analysis of COVID-19 patients and was associated with mild disease and rapid viral clearance^6^. Similarly, evidence from Middle Eastern Respiratory Syndrome (MERS)-CoV and SARS-CoV-1 suggests that the specific T-cell response is critical for recovery and long-term protection^7,8^. After SARS-CoV-2 infection, 100% of recovered patients showed CD4^+^ and 70% CD8^+^ T-cell responses to SARS-CoV-2 and cross-reactive peptides^9^. In adults, SARS-CoV-2-specific CD4^+^ and CD8^+^ T-cell immune memory persists for at least one year after recovery^10–12^, but in children only little data exists on the longevity and protective role of the SARS-CoV-2-specific T-cell response against reinfection and severe disease^13–15^.

Differences in SARS-CoV-2-specific T-cell immunity between mildly affected children and adults have rarely been investigated^16^. Persistent T-cell immunity has been suggested even after mild infection; however, data are mainly available for the adult population^17,18^. Many studies on T-cellular immunity in children have focused on post-vaccination cohorts or those who showed hybrid immunity^19–21^. Difficulties of obtaining suitable samples in children and methodological challenges of cellular assays have led to only a few studies that measured specific T-cell responses to SARS-CoV-2 in children after a single wild-type SARS-CoV-2 exposure^14,15,22^.

Furthermore, conclusive links between clinical symptoms and SARS-CoV-2-specific T-cell responses, as well as potential protection mediated by cross-reactive human coronavirus (HCoV)-specific T-cells remain elusive in children.

Here, we investigated SARS-CoV-2-specific antibody responses and SARS-CoV-2-specific and cross-reactive T-cell responses in longitudinal samples of exposed or convalescent children and adults from a large family cohort (*n* = 68 families)^23^ during the first year after SARS-CoV-2 infection. Humoral response was analyzed using MULTICOV-AB, a multiplex immunoassay with high sensitivity and specificity^24,25^. Cellular responses were analyzed by IFN-γ ELISPOT using four different established epitope compositions (ECs) to discriminate between SARS-CoV-2-specific and HCoV cross-reactive T-cell responses (Extended Data Table 6). Assays were performed with ECs that are recognized exclusively by SARS-CoV-2 convalescent individuals (SI; HLA class I restricted and SII; HLA-DR restricted) and ECs that are cross-reactive to HCoV specific T-cells (CI; HLA class I restricted and CII; HLA-DR restricted)^26^. We correlate humoral and cellular SARS-CoV-2-specific responses with symptoms and report on the role of endemic HCoV antibodies and cross-reactive T-cells. This study simultaneously investigates humoral and cellular immunity following primary SARS-CoV-2 exposure or mild infection within families and provides insights into the long-term persistence of protective immunity to SARS-CoV-2 in children and adults.

## Results

### Study population characteristics

**Fig. 1:**
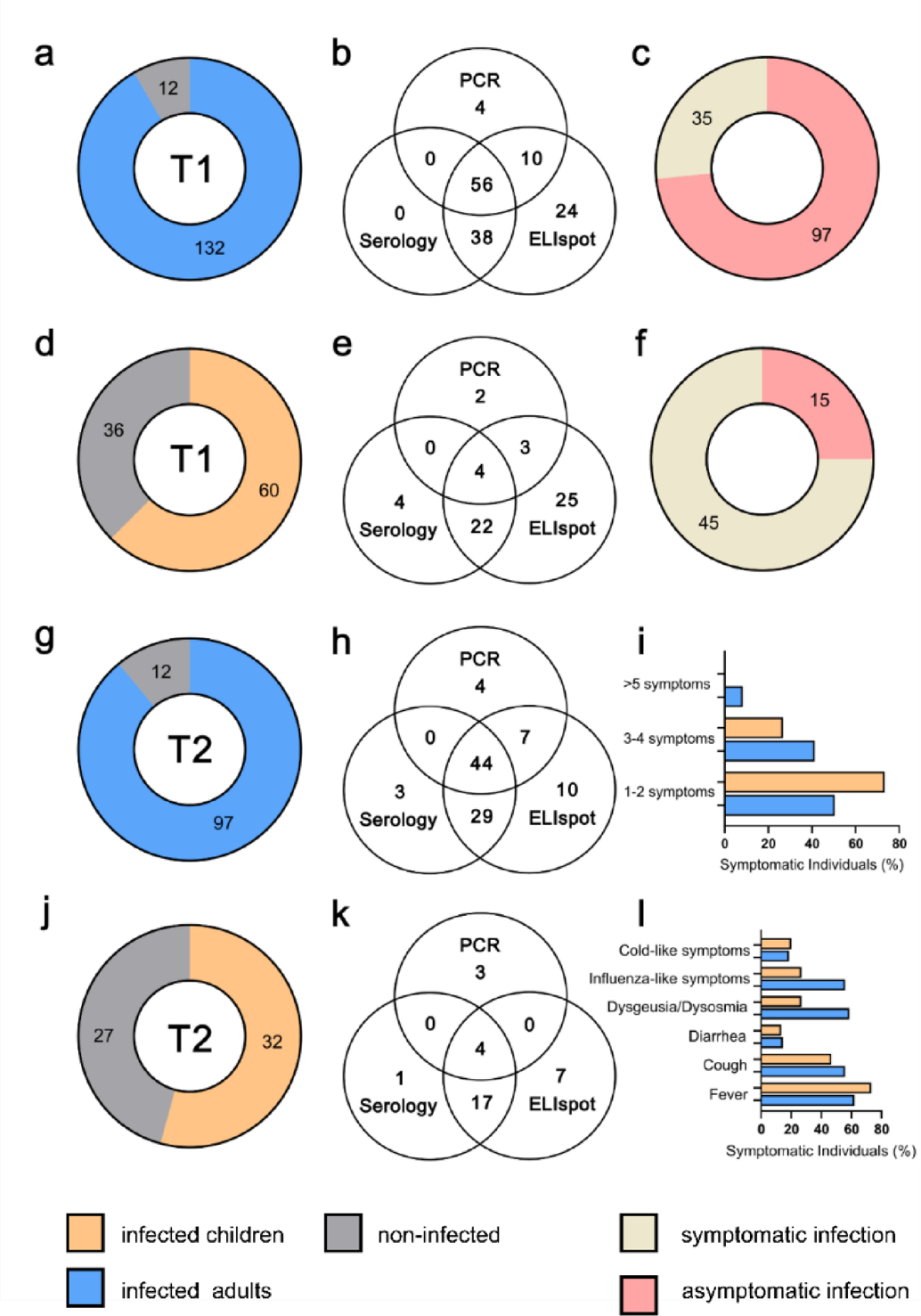
The figure legend is missing, as in all other figures as well. Please check with the original manuscript. Figure legends were included below every figure. Here it should read:Characteristics of the study cohort including proportions of infected and negative individuals (**a**, **d**, **g**, **j**), overlap of PCR positive test results, positive serology results and positive ELISPOT results among infected individuals (**b**, **e**, **h**, **k**), proportions of symptomatic and asymptomatic infections among adults and children (**c**, **f**), cumulative number of symptoms reported (**i**) and frequency of individual symptoms among symptomatic cases (**l**). Left column:: proportions of infected and negative individuals at T1 (**a** and **d**) and T2 (**g** and **j**). Adults (**a** and **g**) within the study cohort had largely been previously infected (blue). Children (**d** and **j**), while also mostly previously infected (orange), also had a larger proportion of negative individuals (grey). Middle column: breakdown of adults (b and h) and children (**e** and **k**) testing positive by PCR, serology and ELISPOT at T1 (**b** and **e**) and T2 **h** and **k**). For adults, the majority tested positive in at least 2 out of 3 tests, whereas children were mostly classified as infected by ELISPOT and/or serology. Right column: Adults (**c**) mostly experienced symptomatic infections (red), whereas children (**f**) were mostly asymptomatic (gold). N’s for both symptomatic and asymptomatic infections are shown. Among symptomatic individuals (i), children (orange) mostly reported experiencing 1-2 symptoms, whereas adults (blue) were evenly split between those who experienced 1-2 or 3-4 symptoms. For individual symptom frequency (h), there were differences in how common certain symptoms were between adults (blue) and children (orange).

Samples were collected at two timepoints: 3–4 months (T1 cohort) and 11–12 months (T2 cohort) after SARS-CoV-2 infection within the family, with 30% lost to follow up (Extended Data Fig. 1). Samples of *n* = 240 individuals (T1 cohort) and *n* = 168 individuals (T2 cohort) were subjected to SARS-CoV-2-specific serology and T cell tests (see Extended Data Methods). Overlap analysis of test results (including PCR) revealed consistency of serology, PCR and ELISPOT test results in the majority of samples. However, we found a substantial proportion of samples testing positive in the SARS-CoV-2-specific ELISPOT only (Fig. 1). Based on the results of these tests, individuals were split into the two groups “infected“ and “negative” for the following statistical analyses. Individuals with a positive PCR result, positive serology test result and/or ELISPOT test result were classified as “infected”. Individuals negative in all three tests were classified as negative. By this definition, 62.5% of children and 92.7% of adults in the T1 cohort were classified as infected (Fig. 1a, d). In the T2 cohort, 54.2% of children and 89.0% of adults were classified as infected (Fig. 1g, j). 3 new infections occurred between T1 and T2, as confirmed by serology and ELISPOT. Demographics and key information for the study cohort are provided in Extended Data Table 1.

**Table 1:** – Characteristics of T-cell only responders, combined individuals and T-cell reverters.

Characteristic	T-cell only responders	Combined	T-cell reverters
n	49	120	15
Adult (n, (%))	24 (49)	94 (78)	7 (47)
Children (n, (%))	25 (51)	26 (22)	8 (53)
Female (n, (%))	25 (51)	59 (49)	9 (60)
Symptoms			
Symptomatic (n, (%))	4 (8)	95 (79)	1 (7)
Asymptomatic (n, (%))	45 (92)	25 (21)	14 (93)
Fever (n, (%))	3 (6)	58 (48)	1 (7)
Cough (n, (%))	2 (4)	49 (41)	1 (7)
Diarrhea (n, (%))	0 (0.0)	14 (12)	0 (0)
Dysgeusia (n, (%))	0 (0.0)	57 (48)	0 (0)
Flu (n, (%))	0 (0.0)	53 (44)	0 (0)
Cold (n, (%))	3 (5)	13 (11)	1 (7)
Cellular Immunity			
SI positive (n, (%))	28 (57)	110 (92)	9 (60)
SII positive (n, (%))	40 (82)	117 (98)	11 (73)
SI and SII positive (n, (%))	21 (43)	107 (89)	6 (40)
Median SI magnitude (IQR)	68 (29–168)	406 (102–904)	18 (3–62)
Median SII magnitude (IQR)	89 (25–345)	1067 (381–1717)	50 (14–148)
HCoVs			
Median CI magnitude (IQR)	34 (9-178)	138 (11-1065)	16 (11–158)
Median CII magnitude (IQR)	225 (66–750)	796 (332–1314)	194 (70–399)
Median OC43 S1 titer (IQR)	0.9 (0.5–1.2)	0.8 (0.5–1.4)	0.8 (0.5–1.2)
Median HKU1 S1 titer (IQR)	0.9 (0.5–1.6)	1.0 (0.5–1.5)	0.8 (0.4–1.7)
Median NL63 S1 titer (IQR)	1.6 (1.0-2.3)	1.4 (0.7–2.5)	1.4 (1.1–1.8)
Median 229E S1 titer (IQR)	0.9 (0.4–1.2)	1.0 (0.6–1.6)	0.8 (0.3-1.0)

T-cell only responders were defined as being ELISPOT positive and PCR and serology negative. Combined individuals were defined as being ELISPOT and serology positive. T-cell reverters were T-cell only responders in T1 but negative in T2. All characteristics were defined using data from T1 only.

Infections in study participants were largely mild or asymptomatic. Asymptomatic infections were far more common in children (75.0%) than in adults (26.5%, Fig. 1c, f). Symptomatic children mostly reported 1–2 symptoms, whereas adults had a greater chance of having multiple (> 3) symptoms (Fig. 1i). Fever was the most common (children 20.0%, adults 44.7%) and diarrhea the least common (children 5.0%, adults 9.8%, Extended Data Table 1) symptom. Among symptomatic individuals, dysgeusia and dysosmia, as well as influenza-like illness were reported more frequently in adults than in children (dysgeusia/dysosmia 59.4% vs. 26.7%, influenza-like illness 56.3% vs. 26.7%, Fig. 1l).

### T-cell only responders – ELISPOT positive and serology negative

Having observed that a large number of adults and children were T-cell positive but serology and PCR negative, we validated the lack of humoral response using four commercial SARS-CoV-2 serological assays, of which 95.8% and 92.0% of adults and children, respectively, tested negative in all four assays (Extended Data Table 2). We then further investigated this group of T-cell-only responders and compared their characteristics to combined humoral and T-cell responders (Table 1). T-cell-only responders were four times more likely to be asymptomatic (92%) than combined responders (21%). Their cellular responses were more frequently only directed against one of the tested specific ECs (SI or SII), with only 43% of individuals testing positive for both SI and SII compared to 89% of combined responders (Table 1). When evaluating the magnitude of T-cell responses compared to negative controls as a measure of abundance of the SI and SII responses (see methods for further details), T-cell-only responders had 6-12-fold reduced magnitude for both SI (median magnitude 68) and SII (median magnitude 89) compared to combined humoral and T-cell responders (SI median magnitude 406, SII median magnitude 1067, Table 1). Of these T-cell responders at T1, 30.6% were negative at T2. This subset was representative of T-cell responders at T1, with no difference in age, gender, or SII magnitude, although SI magnitude was much lower (median SI magnitude 18, see T-cell reverters, Table 1).

### Magnitude of SI/SII response is significantly correlated with humoral status but not titer

**Fig. 2:**
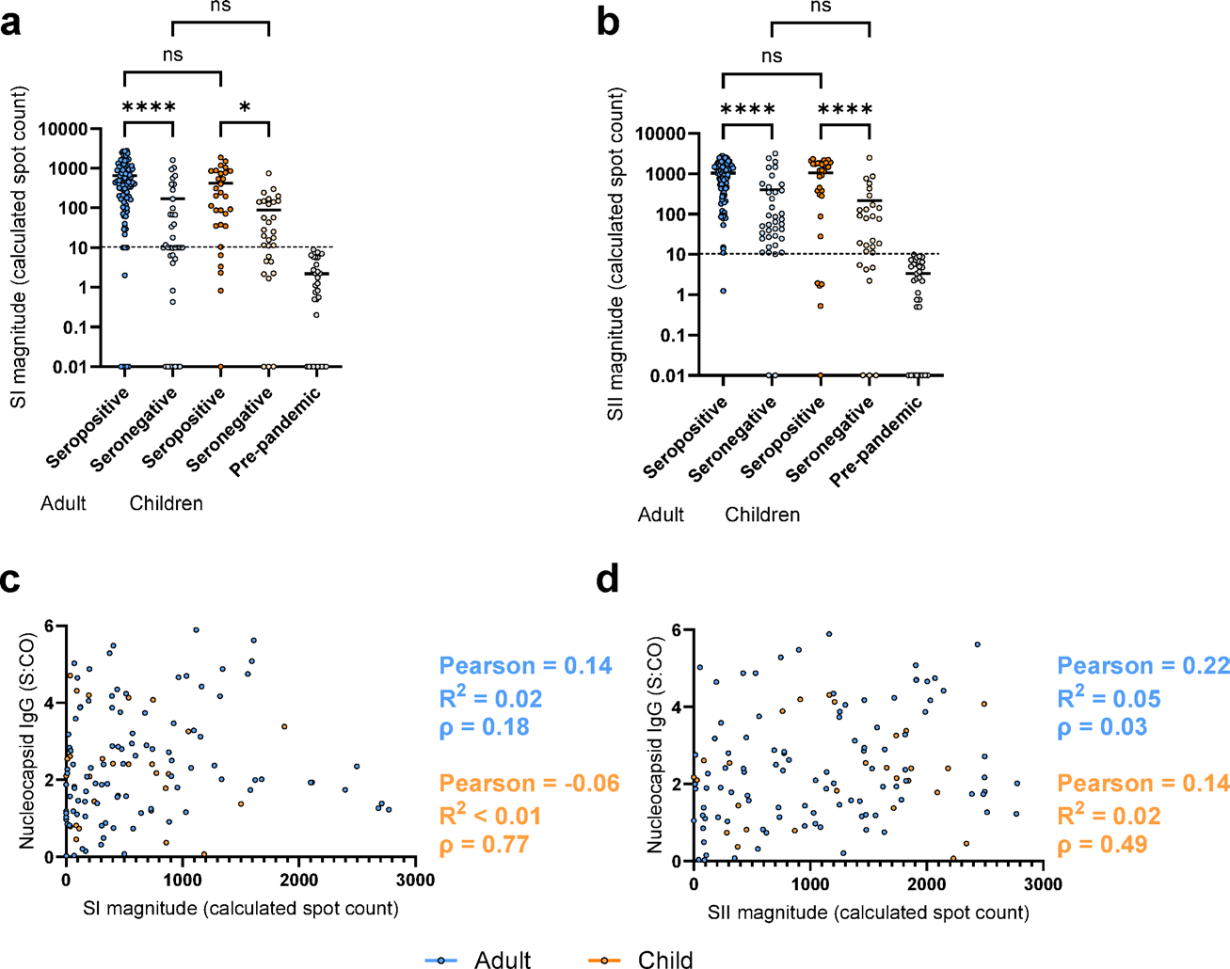
The T-cell magnitude is significantly higher in SARS-CoV-2 seropositive samples than in seronegative samples. SI (**a**) and SII (**b**) magnitude is significantly higher for both adults (blue) and children (orange) in SARS-CoV-2 seropositive samples than in seronegative samples. As a control, pre-pandemic samples (*n*=31) are also included. Scattered dot plots with the line representing the mean. CO value for positivity in the cellular assay is indicated by the dashed line. 0 values were adjusted to 0.01 for display purposes only; all analysis was performed using unadjusted data. Statistical significance was determined by one-way ANOVA (Kruskal-Wallis) with Dunn’s multiple comparison test, with * indicating a p0.05, **** indicating a p0.0001 and ns indicating a p0.05. To evaluate whether the magnitude of T cell response was correlated with titer, SI/SII magnitudes were compared to titers of different SARS-CoV-2 antigens in combined individuals (adults *n*=99, children *n*=28). Combined individuals were used to avoid bias introduced by negative values. Due to the overlap between the peptide pools used for stimulation and the nucleocapsid antigen, the correlations for the nucleocapsid-specific antibodies are shown here (**c** and **d**). Correlation analysis for the other SARS-CoV-2 antigens can be found in Extended Data Table 4 and Extended Data Figure 2. Dot plots showing correlation between SI (**c**) and SII (**d**) magnitude against nucleocapsid-specific antibody titer. Linear regression with Pearson *r* is used to determine the strength of correlation, with Pearson *r*, *r*2 values and p-values provided.  Adults are shown in blue, children in orange.

Adults and children who were SARS-CoV-2 seropositive (Methods) had significantly higher SI (*p* < 0.0001 adults, *p* = 0.01 children, Fig. 2a) and SII (*p* < 0.0001 adults and children, Fig. 2b) magnitudes compared to individuals who were seronegative. However, this increase in SI/SII magnitude was not correlated with an increase in viral nucleocapsid titers for either adults (Pearson 0.14, *p* = 0.18 for SI, Pearson 0.22, *p* = 0.03 for SII) or children (Pearson − 0.06, *p* = 0.77 for SI, Pearson 0.14, *p* = 0.49 for SII, Fig. 2c and d). This lack of significant correlation (*p* > 0.05) was also present for all other SARS-CoV-2 antigens (S, RBD, S1 and S2) for adults and children, except for Adult S2/SI (*p* < 0.001) and S2/SII (*p* = 0.001) (Extended Data Fig. 2 and Extended Data Table 3).

### Correlation of immune response with symptomatic/asymptomatic infection in adults and children

**Fig. 3:**
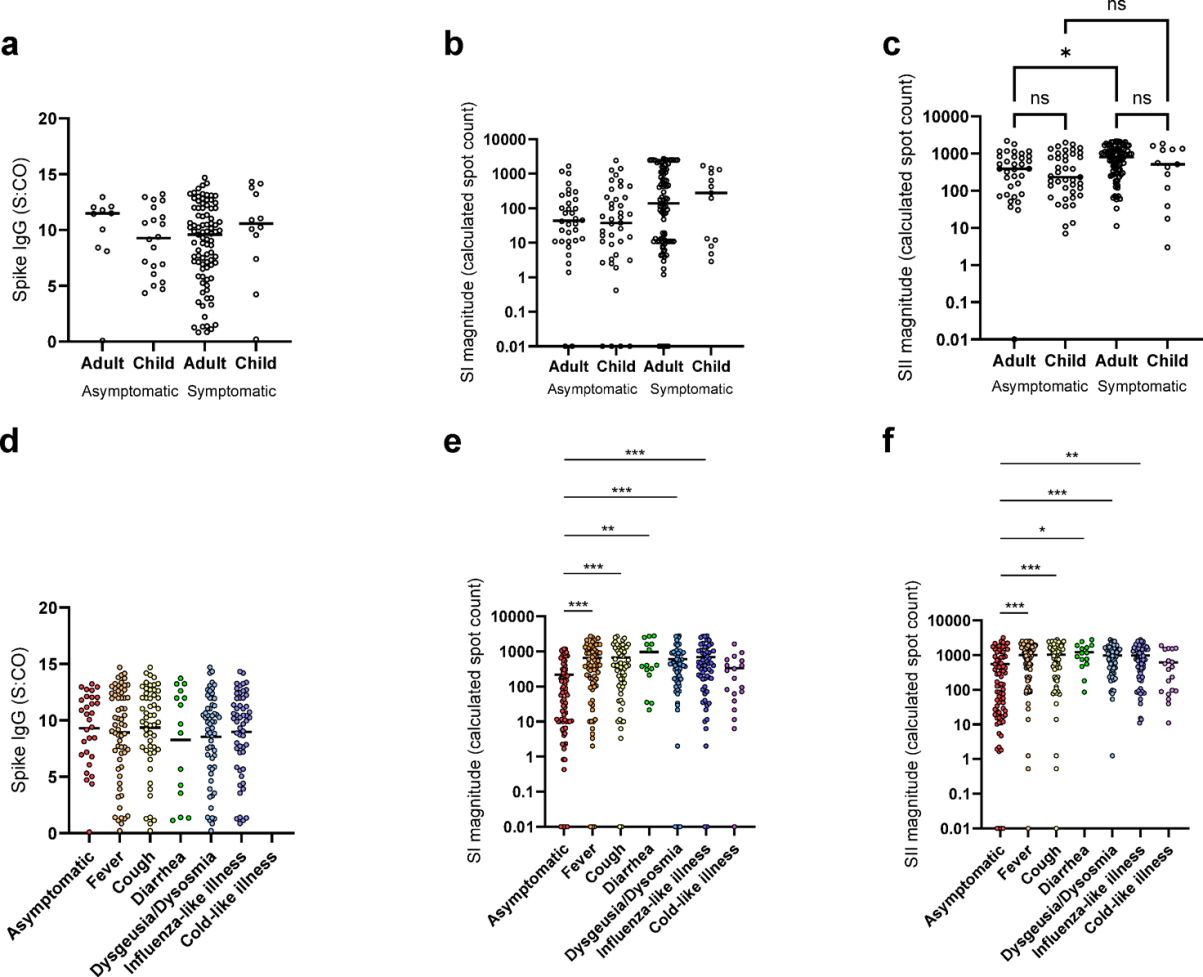
For samples that were SARS-CoV-2 seropositive in the T1 cohort (*n*=131), there was no significant difference in spike-specific IgG titers between individuals with asymptomatic and symptomatic infections (**a**). For samples that were T-cell positive (*n*=182), SI magnitude was also not correlated with symptom status (**b**). For SII magnitude, there was a small significant difference (*p*=0.02) between asymptomatic and symptomatic adults (**c**). When examining individual symptoms (**d**-**f**), there was no difference in spike-specific IgG titers between different symptoms (**d**), whereas there were significant differences in SI (**e**) and SII (**f**) magnitudes between symptoms (**a**-**f**). Scattered dot plots with the line representing the mean. 0 values were adjusted to 0.01 for display purposes only. All analysis was performed using unadjusted data (**a**-**c**). Statistical analysis was Mann-Whitney-U with **** indicating a p0.0001 and ns indicating a p0.05. (**d**-**f**) Statistical analysis was two-way ANOVA (Kruskal-Wallis) with Dunn’s multiple comparisons test. ns indicates a p0.05, * indicates a p0.05, ** indicates a p0.01 and *** indicates a p0.001. Only significant interactions are shown.

Humoral responses were not correlated with symptoms in either adults or children (Fig. 3a). While SI magnitude was also not correlated with symptom status (Fig. 3b), there was a small significant difference (*p* = 0.02) for SII magnitude between asymptomatic and symptomatic adults (Fig. 3c). When evaluating individual symptoms in the entire cohort, we saw significant differences in both SI and SII magnitudes for all symptoms except cold-like illness compared to asymptomatic infections (Fig. 3d-f, Extended Data Table 4).

### Household infection status

**Fig. 4:**
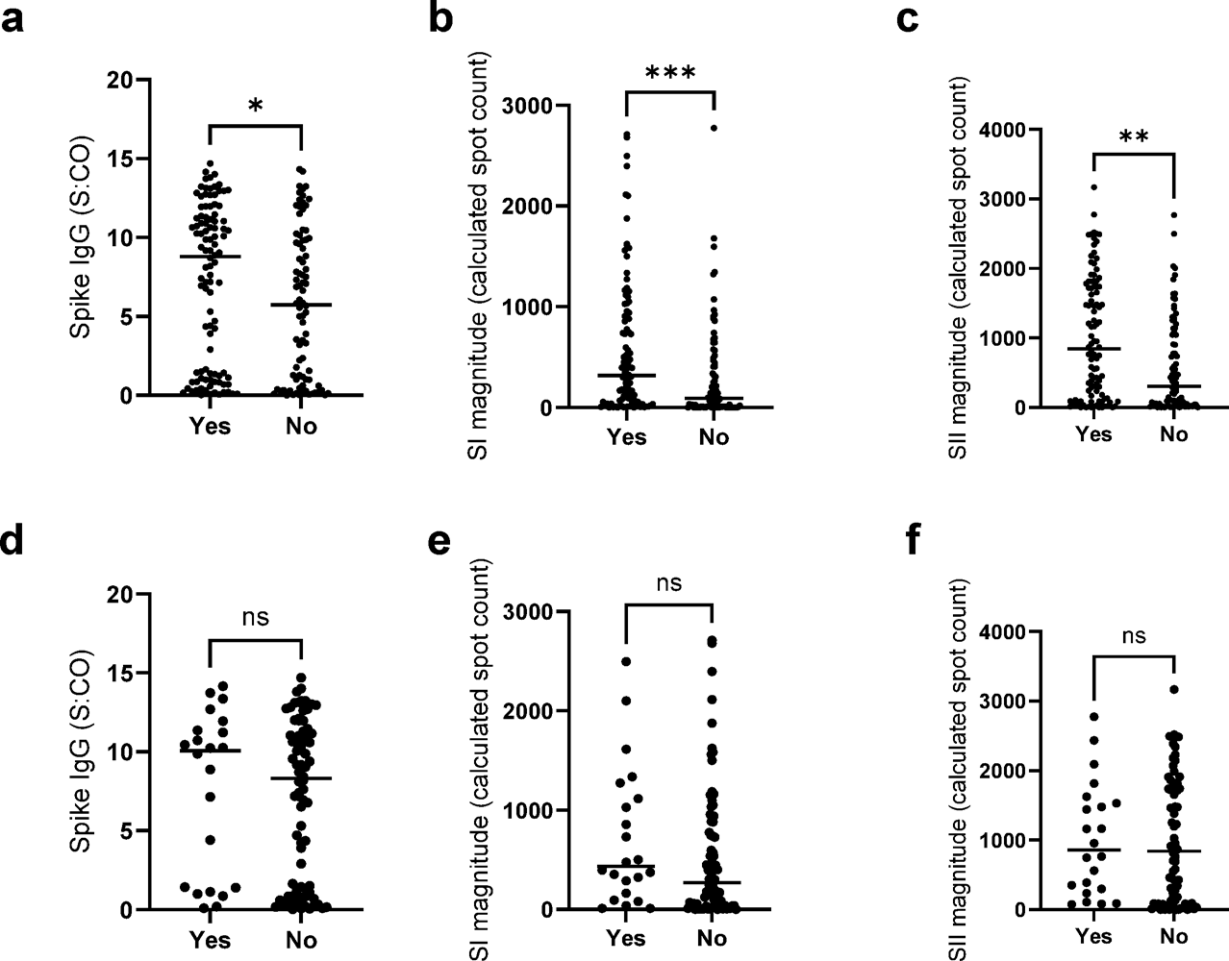
Humoral and cellular titers/magnitudes were significantly increased in households where all members were infected (“yes”), than in those households where only some members were infected (“no”, **a**-**c**). In households where all members were infected, no differences were seen between households where all infections were symptomatic (“yes”) versus where only some infections were symptomatic (“no”, d-f). Scattered dot plots with the line representing the mean. Statistical analysis was Mann-Whitney U with * indicating a p0.05, ** indicating a p0.01 and *** indicating a p0.001.

To assess whether household infection was a factor in humoral and cellular responses, we initially compared humoral and cellular responses between households where all members became infected versus where only some were infected (Fig. 4). Both the humoral titer (*p* = 0.03) and SI (*p* = 0.0003) /SII (*p* = 0.002) magnitudes were significantly increased in households where all members were infected (Fig. 4a-c). However, this was not linked to symptomatic infections, as there was no significant difference in humoral (*p* = 0.56) or cellular response (SI *p* = 0.13, SII *p* = 0.71) between households where all infections were symptomatic versus households with asymptomatic infections too (Fig. 4d-f).

### SARS-CoV-2 infection elicits an increase in cross-reactive T-cell but not humoral response

**Fig. 5:**
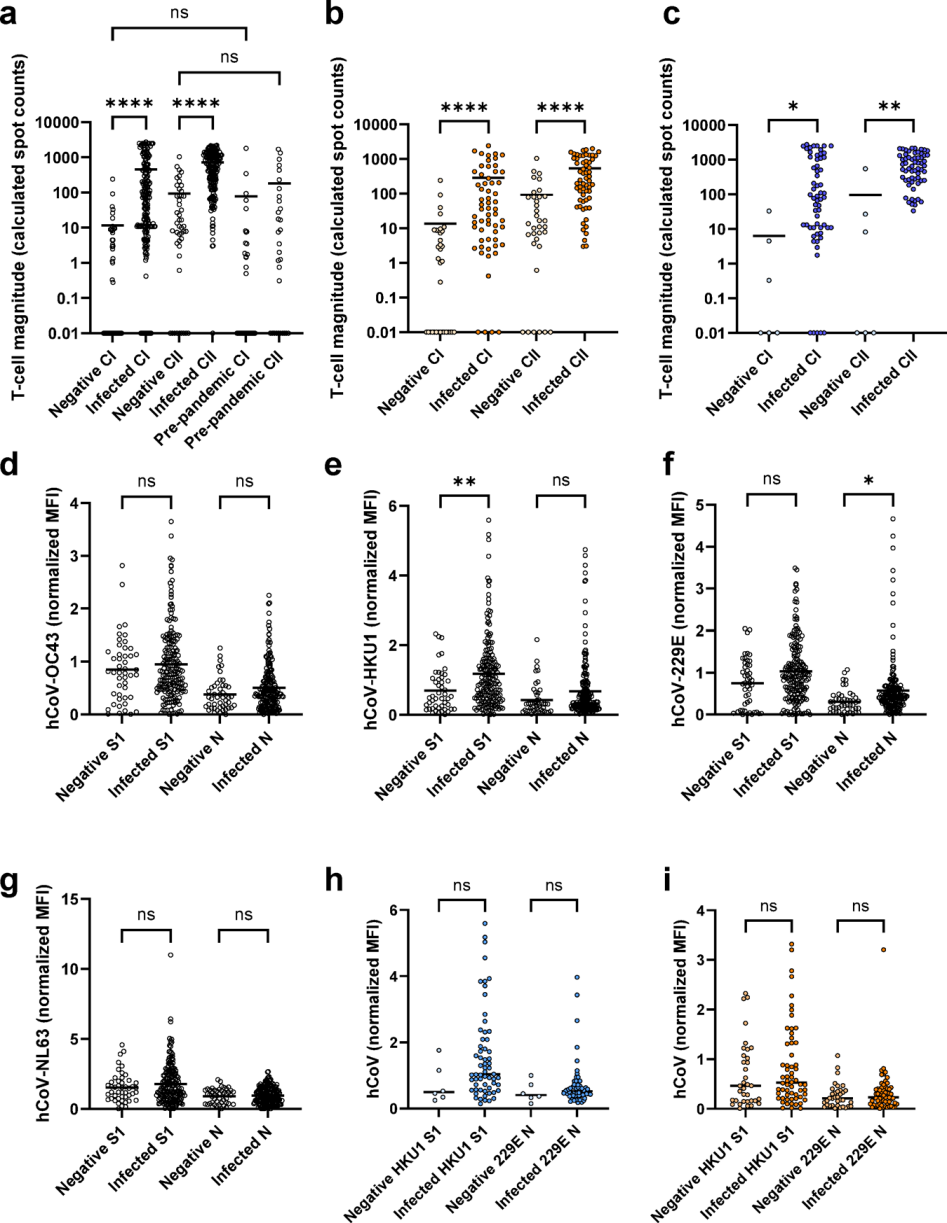
Infection with SARS-CoV-2 increases cross-reactive T-cell response towards the endemic coronaviruses. Humoral and T-cell responses towards the endemic coronaviruses were assessed in the T1 cohort (n=239, 1 sample was not analyzed for cross-reactive T cell responses). As a control, pre-pandemic samples were also included (*n*=31). Endemic coronavirus cross-reactive T-cell response magnitudes (both CI and CII) (**a**) were significantly higher for infected individuals (*n*=192) than for negative individuals (*n*=47), while there was no significant difference for either CI or CII magnitude between negative and pre-pandemic individuals. To avoid bias due to increased exposures and reinfections with increasing age, we corrected for age, identifying the same significant differences among children only (**b**, negative *n*=35, infected *n*=60) and adults aged 40-50 (**c**, negative *n*=6, infected *n*=61). In contrast, there were mostly non-significant differences in titer against either the S1 or N for HCoVs OC43 (**d**), HKU-1 (**e**), 229E (**f**) and NL63 (**g**) between infected and negative individuals, with only HKU1 S1 and 229E N having significant differences (S1 p=0.01, N p=0.03). However, when age is corrected for in the analysis, there are no significant differences in either children (**h**) or adults aged 40-50 (**i**). Scattered dot plots with the line representing the mean. 0 values were adjusted to 0.01 for display purposes only; all data analysis used unadjusted data. Statistical analysis is Kruskal-Wallis with Dunn’s multiple comparison test, with ns indicating a p0.05, * indicating a p0.05 and **** indicating a p0.0001.

Next, we investigated reactive immunity to endemic HCoVs in our study cohort. To do this, we compared magnitudes of T-cell responses following stimulation with HCoV cross-reactive epitope compositions (ECs CI and CII) (Extended Data Table 5) and S1 and Nucleocapsid titers towards the HCoVs OC43, HKU1, NL63 and 229E, in SARS-CoV-2 infected individuals and negative individuals in the T1 cohort. Infected individuals had significantly higher CI (*p* < 0.0001) and CII (*p* < 0.0001) magnitudes, while there was no significant difference in either CI or CII between negative individuals and pre-pandemic individuals (Fig. 5a). In contrast, differences in humoral responses for the HCoVs between infected and negative individuals were largely non-significant (Fig. 5d-g), except for HCoV-HKU1 S1 and 229E Nucleocapsid. Since HCoV exposure is linked to age and there was a significant age difference between the two groups (non-infected median age 11 years, infected median age 39 years), we repeated the same analysis but only examined children (Fig. 5b and i) and adults aged 40–50 years (Fig. 5c and h). When accounting for age, differences in T-cell magnitudes remained statistically significant for children (both *p* < 0.0001, Fig. 5b) and adults (*p* = 0.03 and *p* = 0.003, Fig. 5c). However, there were no longer statistically significant differences for HCoV-HKU1 or 229E in children (Fig. 5h) or adults (Fig. 5i).

### Cellular responses and humoral responses decline similarly in adults and children

**Fig. 6:**
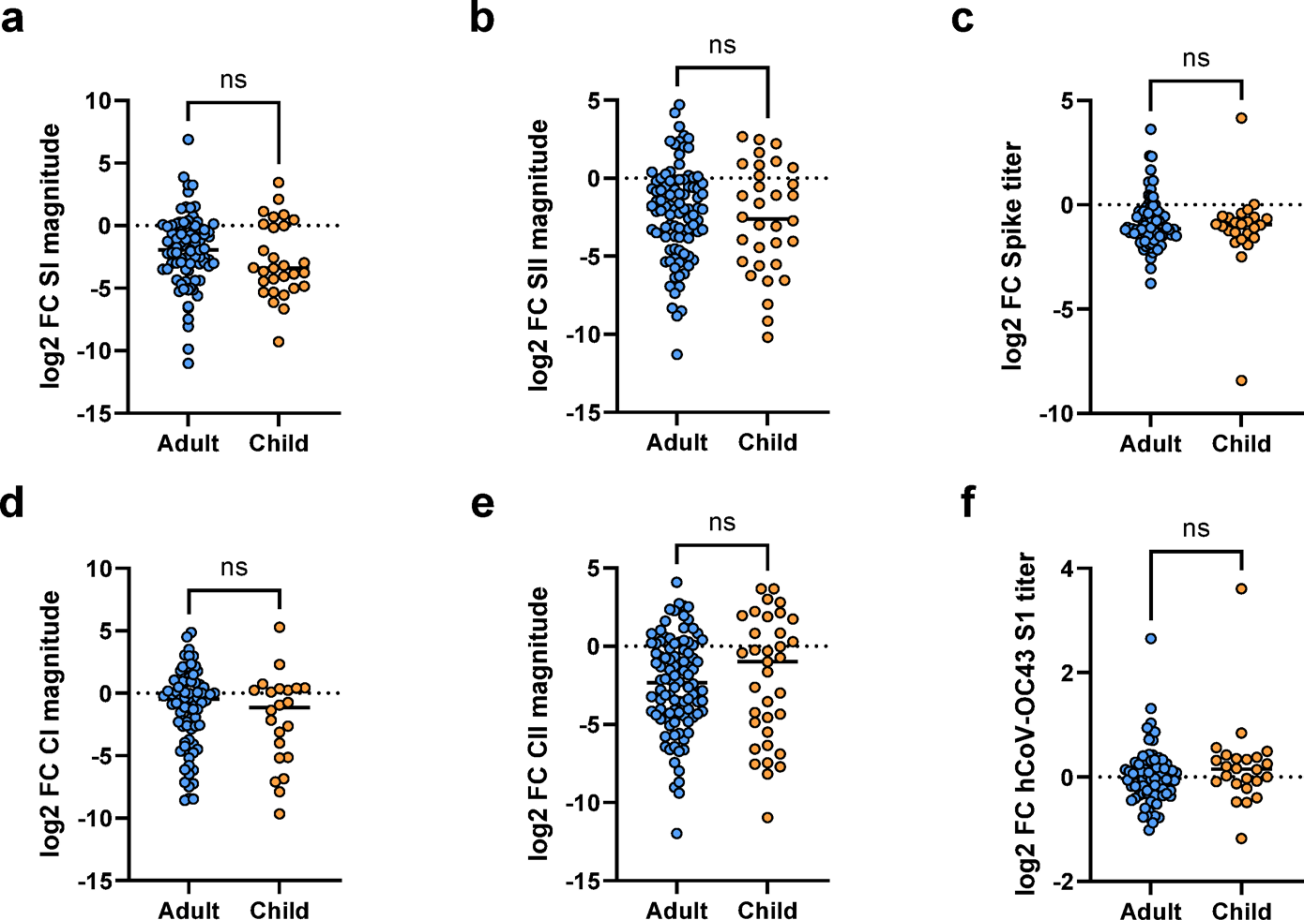
Adults and children have the same decrease in magnitude/titer for both cellular (adult *n*=99, children *n*=36) (**a**-**b**, **d**-**e**) and humoral responses (adult *n*=79, children *n*=24) (**c** and **f**) for both SARS-CoV-2-specific  (**a**-**c**) and HCoV cross-reactive responses (**d**-**f**). Only study participants who are in both the T1 and T2 cohort were included. Log2 fold change (log2 FC) was used to calculate the change in magnitude/titer between T1 and T2. Scattered dot plots with the line representing the mean. Statistical analysis was Mann-Whitney U with ns indicating a p0.05.

**Fig. 7:**
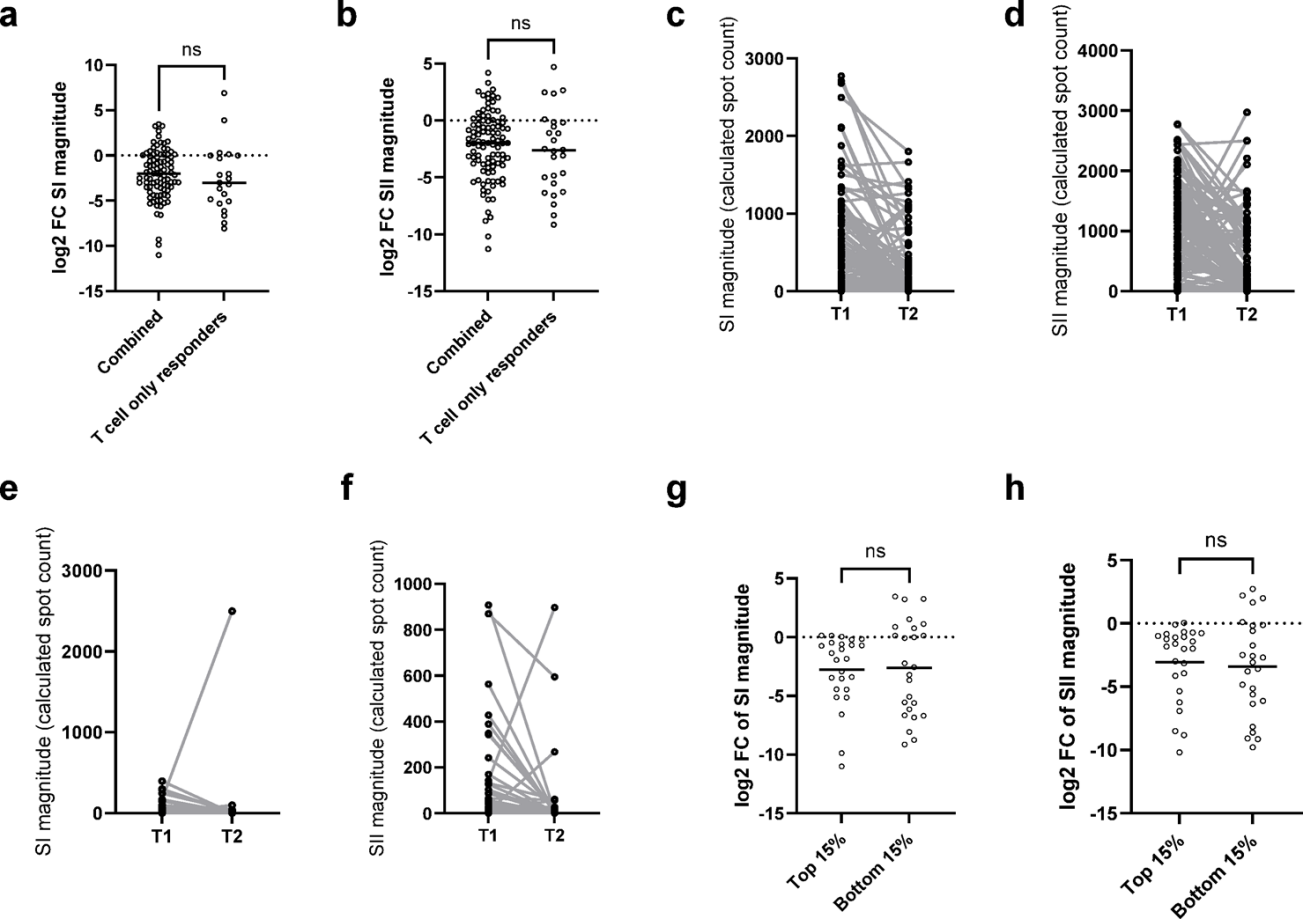
There was no significant difference in decrease in either SI (**a**) or SII (**b**) magnitude between combined individuals (*n*=95) and T cell responders (*n*=30). Change in magnitude was calculated by log2 fold change, from T1 to T2 is then displayed as a scattered dotplot for SI (**a**) and SII (**b**). Scattered dot plots with the line representing the mean. Statistical analysis was Mann-Whitney U with ns indicating a p0.05. To depict variation within the data, line graphs showing change in SI (**c** and **e**) and SII (**d** and **f**) magnitude from T1 to T2 for combined (**c** and **d**) and T cell responders (**e** and **f**). Lines show paired samples. To confirm this was not affected by magnitude at T1, we repeated this analysis for the top 15% and bottom 15% of samples (ranked by SI and SII magnitudes respectively, both n=25). Change in magnitude was calculated as above and displayed as a scattered dotplot for SI (**g**) and SII (**h**). Statistical analysis was Mann-Whitney-U with ns indicating a p0.05.

To assess whether the pediatric and adult immune responses decay differently for humoral and cellular responses, we examined changes in titer/magnitude between the T1 and T2 cohort (Fig. 6). The decline in cellular (Fig. 6a and b) and humoral responses (Fig. 6c) was not significantly different between adults and children. We also evaluated the decline of the endemic HCoVs, finding no differences between adults and children (Fig. 6d-f). To investigate whether the decay in cellular response could be responsible for why so many T-cell responders became negative at T2, we assessed the rate of decay for the combined response group versus T-cell responders (Fig. 7). There was no significant difference in the rate of decay, as determined by log2 fold change, for either SI or SII between the two groups (Fig. 7a and b). However, the rates of decay were highly variable, as seen by the large range in fold change (Fig. 7c-f). To confirm that the rate of decay was consistent regardless of antibody status, we compared it in the top 15 and bottom 15% of T-cell responders and observed no significant difference for either SI or SII magnitudes (Fig. 7g-h). Thus, with T-cell responders having lower T-cell response magnitudes (Table 1), the general decay of T-cell response magnitudes from T1 to T2 may account for reversion in T2.

## Discussion

In this prospective cohort study, we longitudinally analyzed immune responses following familial SARS-CoV-2 exposure 3–4 and 11–12 months after initial infection.

Here, we detected a SARS-CoV-2-specific T-cellular response without seroconversion 3–4 months after primary infection in families in nearly twice as many infected children (46.7%) as adults (25.8%). Children were also more likely than adults to have discordant antibody and T-cell responses. Discordant responses have been detected in up to 41% of seronegative individuals^12,27,28^, but findings in family members exposed to SARS-CoV-2 for the first time are limited^12,29^. Interestingly, a discordant immune response without a spike-antigen-specific antibody and memory B-cell but T-cell responses have been shown after SARS-CoV-2 vaccination of patients with multiple sclerosis and disease-modifying therapies that suppress the immune system^30,31^. Previous studies have used whole-antigen viral peptide megapools, which contain a substantial number of peptides from SARS-CoV-2 that are cross-reactive with other HCoVs, and are therefore unable to distinguish between cross-reactive and SARS-CoV-2-specific T-cell responses^26^. The use of specific and cross-reactive ECs used in the current study allows the differentiation of T-cell responses induced exclusively by SARS-CoV-2 infection from those that may be pre-existing and are boosted by SARS-CoV-2. In our cohort of SARS-CoV-2 exposed or infected (PCR proven) individuals, we found a relatively high proportion (43%) of T-cell responders among seronegative family members. Until now, there was no clear explanation for such a phenomenon. However, several hypothesis have been proposed: In occupational exposure to MERS-CoV and SARS-CoV-2, specific T-cell responses without seroconversion are consistent with the concept of cellular sensitization, whereby T-cells clear infection before it is fully established^32,33^. Additionally, abortive infection upon exposure to SARS-CoV-2 has been described before and has possibly been linked to cross-reactive memory T-cell responses^34^. Furthermore, rapid antibody waning after SARS-CoV-2 infection has been observed^35,36^. We can only speculate on the foundations of this finding and cannot exclude a combined effect of cellular sensitization, abortive infection and rapid antibody waning. Our finding of lower SARS-CoV-2-specific T-cell magnitudes in T-cell-only individuals in combination with the short timespan between exposure and sampling, however, favors the concept of abortive infection. Of note, the proportion of combined humoral and cellular responses was similar in children and adults 11–12 months after infection, largely due to a loss of T-cell reactivity at T2 in samples with low SARS-CoV-2-specific T-cell magnitudes at T1.

Current literature suggests a robust and broad immunological memory after SARS-CoV-2 infection with persisting antibody responses and durable polyfunctional CD4^+^ and CD8^+^ T-cells in adult patients^19,37,38^. Retained T-cellular responses 6 months after infection were also observed in children, even in those being seronegative^39^. Furthermore, we confirm that SARS-CoV-2-specific T-cell responses are maintained for at least 1 year after infection^40^, but it remains unclear whether T-cell responses in the absence of detectable SARS-CoV-2-specific neutralizing antibodies can provide long-term immunity against (re-)infection or a severe course of COVID-19. Taken together, our data emphasize that serological testing alone may underestimate prior SARS-CoV-2 (abortive) infections. This challenges findings from previous serology-based epidemiologic studies, including our own work^41^.

The magnitude of T-cell responses was significantly lower in asymptomatic individuals than in those with definite symptoms such as fever, cough, diarrhea, dysguesia/dysosmia or influenza-like illness. This adds to the observation that children are more likely to lose detectable T-cell responses at T2, especially if they are initially asymptomatic and their T-cell response is weak early in the disease. However, this effect was not as apparent when the analysis was separated into children and adults, most likely due to small numbers. Interestingly, when all family members were infected, both humoral titers and cellular magnitudes were significantly increased compared to households where only some members were infected. Our findings are supported by recent findings in an educational cohort of asymptomatic SARS-CoV-2 infected young adults: asymptomatic infection resulted in decreased antibody and T-cell responses to further exposure to SARS-CoV-2 variants or vaccination, compared to initial symptomatic infection^42^.

Heterologous immunity between endemic coronaviruses (HCoVs) causing mostly “common cold” and SARS-CoV-2 has been described^9,26,43^. Here, we observed a significantly higher cross-reactive T-cell magnitude in SARS-CoV-2 infected individuals compared to uninfected individuals. This indicates that SARS-CoV-2 infection either boosts pre-existing cross-reactive memory T-cell responses or generates these responses *de novo*. However, this was not seen for the humoral response, indicating that the boost in cross-reactive immunity following SARS-CoV-2 infection in both children and adults is limited to T-cells only.

SARS-CoV-2-specific T-cells have been reported to decline with a half-life of 90–200 days^37,43^. In our study, the dynamics of SARS-CoV-2-specific and cross-reactive T-cell magnitudes were similar in children and adults. This suggests that the higher proportion of T-cell negative samples in children was due to a generally lower T-cell response rather than caused by rapid antibody waning or an enhanced decay within one year after infection.

Limitations of our study include the potential recall bias inherent in retrospective self- or parent-reporting of symptoms via questionnaires and physician interviews. In addition, PCR testing for SARS-CoV-2 in Germany at the time of this study was mostly limited to the familial index case when this study was conducted. Furthermore, only a subset of the cohort could be sampled at both study visits, and young children were underrepresented due to reluctance to donate blood and small sample volumes. Overall, humoral and cellular immunity after SARS-CoV-2 infection results from dynamic processes, e.g. it is influenced by age, the time after infection, viral evolution and multiple other factors. Cohort composition, e.g. the ratio of PCR-positive/seropositive and exposed individuals, may influence the detection of SARS-CoV-2-specific T-cellular responses without seroconversion. Furthermore, in the absence of Ct values for the PCR positive/seronegative samples, we cannot completely exclude that they are PCR false positives. Therefore, caution should be exercised when comparing and extrapolating our results to other viral variants (e.g. Omicron) and cohorts, including subcohorts of our own group^44^. To confirm our observations, larger familial cohorts need to be analyzed.

Finally, we did not assess multiple cytokines or T-cell function, nor did we analyze ex vivo T-cell responses. Furthermore, the applied epitope compositions were initially developed based on testing in a large cohort of PCR-confirmed infected adult individuals. Since pre-pandemic pediatric PBMC samples are scarce, we were not able to validate the ECs in such a cohort. Thus, we cannot exclude the possibility that cross-reactive T-cell immunity is divergent in children and adults. This includes both possibilities of either a less diverse T-cell repertoire towards HCoVs due to fewer preceding infections, or a more diverse T-cell repertoire due to more recent primary HCoV infections in children. Similarly, the T-cell repertoire towards HCoVs in adults may become more focused on immunodominant epitopes with repeated exposure. Previously, the choice of peptides and duration of stimulation may have led to reduced sensitivity and consequently missed the detection of individuals with a SARS-CoV-2-specific T-cellular response without seroconversion. However, the calculated T-cell magnitude values used in some of the analyses have certain limitations. First, SI/CI response values have limited inter-individual comparability, because the number of HLA-matched peptides within the ECs is dependent on individual HLA type. Second, HLA typing of individuals has not been conducted and thus, some individuals testing negative for SI or CI may be explained by complete mismatch of HLA type and epitope composition. Values of the SII/CII responses have greater comparability due to the promiscuity of HLA-DR-binding peptides^17^. These comparability issues are compensated for when analyzing effects in large cohorts such as ours.

The strength of our study is that we evaluated a longitudinal familial cohort of previously SARS-CoV-2-naïve children and adults that were exposed to or infected with SARS-CoV-2 using a combination of a highly accurate multiplex immunoassay for both endemic and pathogenic coronaviruses and a highly specific IFN-γ ELISPOT assay in the early phase of the pandemic, i.e. before SARS-CoV-2 vaccines were available. Thus, our study provides immunological characterization and real-world data representative of the majority of SARS-CoV-2 infections within children and helps to fill the gap in understanding the specific T-cell response to SARS-CoV-2 in asymptomatic or pauci-symptomatic children^45^.

In summary, our data indicate that a SARS-CoV-2-specific T-cell response without detectable antibodies is possible in intrafamilial exposure. We hypothesize that exposed children are more likely than adults to mount such a discordant T-cell response, reflecting either cellular sensitization or abortive infection with rapid viral clearance. Consequently, epidemiologic data based solely on SARS-CoV-2 antibodies may underestimate prior viral exposure, particularly in children. SARS-CoV-2-specific T-cell responses correlated with symptoms, and infection boosted cross-reactive T-cell but not humoral responses. SARS-CoV-2-specific T-cell and antibody responses were detected in most mildly affected individuals one year after infection, supporting equally persistent humoral and cellular immunity to SARS-CoV-2 in children and adults.

## Materials and methods

### Study cohort

This study was part of a non-interventional, prospective observational national multi-center cohort study, including 163 children and 245 adults from 107 families in Baden-Württemberg, Germany^23^. Participants were recruited during the first wave of the pandemic via local health authorities and an in-hospital database of familial households with at least one laboratory-confirmed SARS-CoV-2 infection. During this time, strict infection prevention and control measures were in place in the public and hospital settings and included wearing face masks and restrictions on social contact (closing kindergartens and schools, canceling events, restrictions regarding spending time out of doors, closure of bars and restaurants etc^46^).

For this sub-study, peripheral blood mononuclear cells (PBMCs), sera and demographic and clinical data were collected at the study site in Tübingen only (see Extended Data for further details on cohort, data collection and data variables). Ethics approval was obtained from the independent ethics committee of the Medical Faculty, University of Tübingen (293/2020BO2). Written informed consent was obtained from adult participants and from parents or legal guardians on behalf of their children at both sampling time points. Children’s preferences on whether to provide a blood sample were respected throughout. This study was registered on the German Clinical Trials Register (DRKS), study ID 00021521, conducted according to the Declaration of Helsinki, and designed, analyzed and reported according to the Strengthening the Reporting of Observational Studies in Epidemiology (STROBE) reporting guidelines. The full study protocol can be found at https://www.drks.de/drks_web/navigate.do?navigationId=trial.HTML&TRIAL_ID=DRKS00021521. Samples were collected at two timepoints: T1 at a median of 117 days (IQR 108–123) after earliest symptom onset within the family, and T2 at 346 days (IQR 338–354) post-symptom onset. Overall, 408 samples (240 at T1, 168 at T2) were collected from 240 individuals (168 individuals donated samples at both T1 and T2).

Eligibility criteria can be found in Extended Data Methods, with the T1 and T2 cohorts shown as Extended Data Fig. 1.

Individuals were defined as infected on the basis of having a positive PCR test, a positive serological test result (IgG positive with MULTICOV-AB) and/or a positive SARS-CoV-2-specific T-cell response (SI - specific HLA class I EC or SII – specific HLA-DR EC or both positive in IFN-γ ELISPOT). Infected individuals were further subclassified for certain analysis on the basis of their test results. “Combined” individuals were positive for both serology and T-cell response. This group resembles complete infection. “Seropositive” individuals were positive for serology only. “T-cell responders” were positive for T-cell response only. This group resembles abortive infection.

### Laboratory analysis

Humoral responses against SARS-CoV-2 in all samples were analyzed using MULTICOV-AB, a previously published bead-based multiplex immunoassay that simultaneously analyses antibody binding to 23 antigens from SARS-CoV-2 (including VOCs) and the endemic coronaviruses (e.g. HCoV-OC43)^24,47^. A full list of antigens included within this study can be found in Extended Data Table 6. Samples are classified as being positive using a dual S and RBD cut-off. Samples were also analyzed using four commercially available antibody assays from EuroImmun (EuroImmun-Anti-SARS-CoV-2 S1 ELISA IgG and IgA), Siemens (Siemens Healthineers SARS-CoV-2 RBD IgG) and Roche (Elecsys Ig Nucleocapsid pan-Ig). All assays were performed as per the manufacturer’s instructions. Serological analysis of the samples used within this study has been previously published^25^.

Cellular responses were assessed using Interferon-γ (IFN-γ) ELISPOT as described previously^26^. Briefly, PBMCs were thawed, counted and seeded in 48-well plates in concentrations of 1 × 10^7^ cells ml^-1^ in IMDM medium (Sigma) supplemented with 10% human serum (Sigma), 50 µM β-mercaptoethanol (Roth) and 1% peniciline / streptomycine (Sigma) at day 0. Human Leucocyte Antigen (HLA) class I or HLA-DR restricted epitope compositions (EC) (1 µg ml^-1^ per peptide for HLA class I restricted peptides and 5 µg ml^-1^ per peptide for HLA-DR restricted peptides) were added to the PBMC cultures at day 1 (see Extended Data Table 5 for peptide sequences and ECs)^19^ Cultures were supplemented with 10 U ml^-1^ interleukin-2 (Novartis) and 10 ng ml^-1^ interleukin-7 (PeproTech) at days 2, 5, 7 and 9. PBMCs were harvested and counted at day 12 and analyzed by interferon-γ enzyme-linked immunospot (IFN-γ ELISPOT) assay in replicates (2–5, per sample depending on cell counts, except for positive controls; 1–2). PBMCs were seeded in 96-well microtiter plates (Millipore) coated with anti-IFN-γ (clone 1-D1K, 2 µg ml^-1^, MabTech) at 2–5 × 10^5^ cells per well. ECs for HLA class I (1 µg ml^-1^ per peptide) and HLA-DR (5 µg ml^-1^ per peptide) were added to each well. Negative controls consisted of equimolar amounts of 10% DMSO. PHA (10 µg ml^−1^, Sigma-Aldrich) served as positive control. After 24 h incubation, cells were removed and wells were washed three times with PBS / 0.05% Tween20 for the processing of ELISPOT plates. IFN-γ spots were labeled by incubation with anti-IFN-γ biotinylated detection antibody (clone 7-B6-1, 0.3 µg ml^−1^, MabTech) for 2 h and visualized by addition of ExtrAvidin-alkaline phosphatase (1:1,000 dilution, Sigma-Aldrich) and substrate BCIP/NBT (5-bromo-4-chloro-3-indolyl-phosphate/nitro-blue tetrazolium chloride, Sigma-Aldrich). The reaction was stopped after 7 min, plates were dried, and spot counts were determined using an ImmunoSpot S6 Ultra-V Analyzer (C.T.L.). Mean spot counts of replicates were calculated and normalized to 5 × 10^5^ cells. Magnitudes of T-cell responses were calculated by subtraction of normalized mean spot counts of EC-stimulated samples and respective negative controls. T-cell responses were considered as positive if (I) the normalized mean spot count value was at least 3-fold above the respective negative control and (II) the magnitude value was ≥ 10. Normalized mean spot count values of the positive control had to be ≥ 100. Exemplary ELISPOT assay pictures derived from *n* = 10 individuals (5 adults and 5 children) who were antibody-negative and T-cell positive at T1 are shown in Extended Data Fig. 3. As a further control, 31 pre-pandemic samples were analyzed to confirm assay performance. A comparison of SI and SII magnitude between samples from T1 with a PCR-confirmed infection and these pre-pandemic samples is included as Extended Data Fig. 4.

### Data analysis

Initial data collection was done using secuTrial^®^ electronic data capture system. Formal data analysis was performed on GraphPad Prism (version 9.3.1). The exact number of individuals and any exclusion/inclusion criteria for analysis is indicated within the appropriate figure legend. Statistical analyses performed are described in the figure legends. P values < 0.05 were considered to be significant.

## Electronic supplementary material

Below is the link to the electronic supplementary material.

Supplementary Material 1

## Data Availability

The datasets used and/or analysed during the current study are available from the corresponding author on reasonable request.
