# Supplementary material for: Longitudinal analysis of humoral and cellular immunity in SARS-CoV-2 exposed families
Source: Sci Rep. 2025 Jul 18;15:26041. doi: 10.1038/s41598-025-07739-3 (PMC12274566; doi:10.1038/s41598-025-07739-3)
Supplement: Supplementary file 1 — Supplementary Material 1 [file 41598_2025_7739_MOESM1_ESM.docx]

**Longitudinal analysis of humoral and cellular immunity in SARS-CoV-2 exposed families**

Alex Dulovic Dr.rer.nat.^1*^, Armin Rabsteyn Dr.rer.nat.^2,3,4*^, Jonathan Remppis MD^2^, Irene K. E. Gentzcke^2^, Julia Mueller^2^, Nadja Tuecks^2^, Matthias Becker Dr.rer.nat^1^, Daniel Junker M.Sc^1^, Philipp D. Kaiser Dr.rer.nat^1^, Bjoern Traenkle Dr. rer.nat^1^, Ulrich Rothbauer Dr.rer.nat^5^, Juliane S. Walz MD^3,4,6,7^, Andreas Peter MD^8^, Sebastian Hörber MD^8^, Tina Ganzenmueller MD^9^, Thomas Iftner PhD^9^, Maximilian Stich MD^10,11,12,13^, Burkhard Tönshoff MD^10^, Philipp Henneke MD^14,15^, Roland Elling MD^14,15^, Klaus-Michael Debatin MD^16^, Ales Janda MD^16^, Nicole Schneiderhan-Marra Dr.rer.nat^1^, Axel R. Franz MD^2,17^, Peter Lang MD^2,3,$^ and Hanna Renk MD^2,17,$,#^

1 – NMI Natural and Medical Sciences Institute at the University of Tübingen, Reutlingen, Germany

2 – University Children’s Hospital Tübingen, Tübingen, Germany

3 – German Cancer Consortium (DKTK) and German Cancer Research Center (DKFZ), Partner Site Tübingen, Tübingen, Germany

4 - Cluster of Excellence iFIT (EXC2180) “Image-Guided and Functionally Instructed Tumor Therapies”, University of Tübingen, Tübingen, Germany

5 – Pharmaceutical Biotechnology, University of Tübingen, Tübingen, Germany

6 - Department of Peptide-based Immunotherapy, Institute of Immunology, University and University Hospital Tübingen, Tübingen, Germany

7 - Clinical Collaboration Unit Translational Immunology, German Cancer Consortium (DKTK), Department of Internal Medicine, University Hospital Tübingen, Tübingen, Germany

8 – Institute for Clinical Chemistry and Pathobiochemistry, University Hospital Tübingen, Tübingen, Germany

9 – Institute for Medical Virology and Epidemiology of Viral Diseases, University Hospital Tübingen, Tübingen, Germany

10 – Heidelberg University, Medical Faculty, Department of Pediatrics I, University Children‘s Hospital Heidelberg, Germany

11 – Department of Infectious Diseases, Molecular Virology, University of Heidelberg, Heidelberg, Germany

12 Division of Virus-Associated Carcinogenesis, German Cancer Research Center, Heidelberg, Germany

13 German Center for Infection Research, Heidelberg Partner Site, Heidelberg, Germany

14 Center for Pediatrics and Adolescent Medicine, Medical Center Freiburg, Germany and Faculty of Medicine, University of Freiburg, Freiburg, Germany

15 – Institute for Immunodeficiency, University Medical Center Freiburg, Freiburg, Germany

16 - Department of Pediatrics and Adolescent Medicine, Ulm University Medical Center, Ulm University, Ulm, Germany

17 – Center for Pediatric Clinical Studies, University Hospital Tübingen, Tübingen, Germany

*These authors contributed equally and share first authorship

^$^ shared last authorship

# corresponding author

**Correspondence**

Hanna Renk, MD

University Children’s Hospital Tübingen

Hoppe-Seyler-Str 1

72076 Tübingen

Phone: +49 7071 29-62250

Email: Hanna.Renk@med.uni-tuebingen.de

**Extended Data Additional Methods**

### Cohort

Each of the families had at least one individual with a SARS-CoV-2 infection confirmed either by reverse-transcriptase polymerase chain reaction (RT-PCR) or a symptomatic and later serologically proven infection. Due to restrictions in obtaining a SARS-CoV-2 RT-PCR test during the first wave (children and asymptomatic contacts of an index case were not tested routinely), serological assays were the only means to identify previous infection. Samples were collected at 2 timepoints: T1 cohort between 11^th^ and 19^th^ of July 2020, approximately 4 months post-infection, and T2 cohort between 20^th^ February and 17^th^ March 2021, approximately 12 months post-infection. The multi-center study (described elsewhere)(*23*) was initiated by the four University Children’s Hospitals of Freiburg, Heidelberg, Tübingen and Ulm in Baden-Württemberg, Germany, and approved by the independent ethics committee of each center.

### Study participants and eligibility criteria

Families were identified during the first wave of the pandemic in Baden–Württemberg, Germany.

Inclusion criteria:

(i) At least one family member (male or female) aged 1–17 years.

(ii) Parents and other adults (male or female) living in the same household with the investigated children (without age limit).

(iii) Residency in the state of Baden-Württemberg.

(iv) Written consent to the study.

Key exclusion criteria:

(i) Severe congenital diseases (e.g. infantile cerebral palsy, severe congenital malformations).

(ii) Congenital or acquired immunodeficiencies.

(iii) Insufficient comprehension of German language.

### Data collection

Children and adults within eligible familial households completed a questionnaire collecting demographic information (e.g. date of birth, gender, height, weight, smoking, chronic conditions, previously PCR-proven SARS-CoV-2 infection), the presence, start date and duration of symptoms (fever, cough, dysgeusia, diarrhea or any other symptom) in temporal association (max. two weeks prior or later) with the onset of the SARS-CoV-2 infection within the family. For younger children, parents provided information on symptoms. Participants also provided EDTA blood and serum samples for immunological analysis at both T1 and T2. Data on vaccination and potential re-infection within the family were collected at T2. We investigated all invited families with at least one child to avoid selection bias. Questionnaires were checked for missing or inadequate data and inconsistencies; where possible, these points were clarified retrospectively with the families.

### Data variables

Individuals were defined as infected on the basis of having a positive PCR test, a positive serological test result (IgG positive with MULTICOV-AB) and/or a positive SARS-CoV-2 specific T cell response (SI or SII or both positive in IFN-γ ELISPOT). Infected individuals were further subclassified for certain analysis on the basis of their test results. “Combined” individuals were both positive for both serology and T-cell response. This group resembles complete infection. “Seropositive” individuals were positive for serology only. “T-cell responders” were positive for T-cell response only. This group resembles abortive infection.

“Symptomatic” infections were defined on the basis of having at least one symptom out of fever, cough, diarrhea, dysgeusia, general flu symptoms or general cold symptoms in addition to being infected. “Asymptomatic” individuals were defined as infected individuals who did not report any of the above symptoms. “Time post symptom onset” was calculated as the number of days between the first date of symptom onset in any infected individual and the sampling date at T1 and T2 respectively.

**Blood sample collection and preparation of PBMCs**

Samples were collected at two separate time points, an early time point (T1) at a median of 117 days (IQR 108-123) after earliest symptom onset within the family and a late time point (T2) at 346 days (IQR 338-354) post-symptom onset. Blood samples were collected by venipuncture from all consenting adults and children within the study. Serum was isolated using separate serum tubes at the day of collection by centrifugation, aliquoted and frozen at −80 °C until further use. Blood samples were collected in EDTA tubes. Samples were centrifuged for 7 min at 1950 g / RT. Plasma samples were collected from the supernatant and cryopreserved until further use. The remaining blood samples were diluted 1:1 with PBS. Samples were layered onto 15 ml of Ficoll-Hypaque cell separation solution (density of 1.077 g/ml) in 50 ml tubes. Samples were centrifuged for 30 min at 800 g / RT w/o brake. PBMC layers were collected, transferred to 50 ml tubes and topped up to 50 ml volume with PBS. Samples were centrifuged for 10 min at 500 g / RT. After two additional washing steps with 50 ml PBS (10 min / 400 g / RT; 10 min / 300 g / RT), cells were counted and cryopreserved in serum-free cell freezing medium until further use.

### Multiplex immunoassay: MULTICOV-AB

Humoral responses against SARS-CoV-2 in all samples were analyzed using MULTICOV-AB, a previously published bead-based multiplex immunoassay that simultaneously analyses antibody binding to 23 antigens from SARS-CoV-2 (including VOCs) and the endemic coronaviruses (e.g. HCoV-OC43) (*17, 36*)). A full list of antigens included within this study can be found as **Extended Data** **Table 9**. MULTICOV-AB analysis of the samples used within this study have been previously published (*18*). Briefly, samples were measured in 384-well plates, with all pipetting steps performed using a Beckmann Coulter i7 pipetting robot. Antigens were coupled by EDC/s-NHS or Anteo coupling to spectrally distinct populations of MagPlex beads (Luminex Technology). Samples were diluted in assay buffer (1:4 Low Cross Buffer (Candor Bioscience GmbH) in CBS (1x PBS + 1% BSA) + 0.05% Tween20) and added to bead mix to a final dilution factor of 1:400, before being incubated for 2 hours at 21 °C on a thermomixer (1500rpm). Unbound antibodies were then removed by washing with Wash buffer (1x PBS, 0.05% Tween20). Bound antibodies were detected using RPE-conjugated human IgG (3 µg/mL, Biozol) or IgA (5 µg/mL, Biozol) by incubation for 45 mins at 21 °C, 1800 rpm on a thermomixer. Following a further washing step, beads were resuspended in 80 µL of washing buffer and shaken briefly for 3 mins at 1500 rpm. Plates were then measured using a FLEXMAP-3D (Luminex, DiaSorin) instrument running xPONENT Software (version 4.3) with the following settings: 60 µL, 80 s timeout, 35 events, Gate 7500-15000 and Reporter Gain: Standard PMT. For quality control, eight wells for each QC sample plus eight blank wells (negative control) were included on each 384-well plate (*36, 37*). Additionally, control beads coupled with human IgG, goat-anti-human IgG, human IgA and goat-anti-human IgA were included in each well to act as controls for both sample addition and signal system addition. To pass QC, each sample had to meet the minimum threshold for number of beads per ID (35), have a sample and signal system control bead value within normal range and pass plate-by-plate QC sample controls. Any plate or sample that failed QC was re-measured (14/576). Normalization values, as a proxy for antibody titer, against each antigen were generated by dividing the raw median fluorescence intensity (MFI) value by the mean plate-by-plate MFI of QC2 (IgG) or QC3 (IgA). For SARS-CoV-2, normalization values >1 for both the trimeric spike and wild-type RBD indicate positivity. To reduce analytical variations, all samples were analyzed in the same run. Serological analysis was performed blinded for all clinical covariables.

**Extended Data Table 1 – Demographics and key information for the study cohort**

|  | T1 cohort | | T2 cohort | |
| --- | --- | --- | --- | --- |
|  | Adult | Children | Adult | Children |
| Number of donors by age group | 144 | 96 | 109 | 59 |
| Median Age – years (IQR) | 44 (37-50) | 10 (7-13) | 45 (39-50) | 10 (7-13) |
| Female (n (%)) | 72 (50.0) | 46 (47.9) | 55 (50.5) | 28 (47.5) |
| BMI (IQR) | 25 (22-28) | 16 (15-19) | 26 (24-29) | 17 (15-19) |
| **SARS-CoV-2 infected individuals (n (%))** | 132 (91.7) | 60 (62.5) | 97 (89.0) | 32 (54.2) |
| SARS-CoV-2 seropositive participants (n (%)) | 99 (75.0) | 32 (53.3) | 76 (69.7) | 22 (68.8) |
| SARS-CoV-2 specific T cell positive participants (n (%)) | 128 (97.0) | 54 (90.0) | 90 (92.8) | 28 (87.5) |
| SARS-CoV-2 PCR positive participants | 70 (53.0) | 9 (15.0) | 55 (56.7) | 7 (21.9) |
| **Symptoms at disease onset (of infected individuals) (n (%))** |  |  |  |  |
| - Asymptomatic (n (%)) | 36 (27.3) | 45 (75.0) | 1. (26.8) | 22 (68.8) |
| - Symptomatic (n (%)) | 96 (72.7) | 15 (25.0) | 71 (73.2) | 10 (31.2) |
| - Fever | 59 (44.7) | 12 (20.0) | 44 (45.4) | 6 (18.8) |
| - Cough | 54 (40.9) | 7 (11.7) | 37 (38.1) | 5 (15.6) |
| - Diarrhea | 13 (9.8) | 3 (5.0) | 10 (10.3) | 2 (6.3) |
| - Dysgeusia/Dysosmia | 57 (43.2) | 4 (6.7) | 46 (47.4) | 3 (9.4) |
| - Influenza-like illness | 54 (40.9) | 4 (6.7) | 40 (41.2) | 2 (6.3) |
| - Cold-like illness | 18 (13.6) | 3 (5.0) | 9 (9.3) | 0 (0.0) |
| **Interval (days) positive PCR test to sample collection (IQR)** | 110 (102-119) | 108 (103-123) | 329 (311-344) | 340 (337-344) |
| **Interval (days) from symptoms onset to sample collection** | 117 (108-124) | 108 (103-117) | 342 (333-351) | 335 (333-343) |
| **Hospitalized (of infected) (n (%))** | 4 (3.0) | 0 (0.0) | 4 (4.3) | 0 (0) |
| **Comorbidities (n (%))** |  |  |  |  |
| - Arterial hypertension | 13 (9.0) | 0 (0.0) | 10 (9.2) | 1. (0.0) |
| - Asthma/Atopy | 19 (13.2) | 9 (9.4) | 13 (11.9) | 6 (10.2) |
| - Cardiac insufficiency | 4 (2.8) | 0 (0.0) | 3 (2.8) | 1. (0.0) |
| - Chronic pulmonary disease | 0 (0.0) | 0 (0.0) | 0 (0.0) | 1. (0.0) |
| - Diabetes mellitus | 4 (2.8) | 0 (0.0) | 3 (2.8) | 0 (0.0) |
| - Disorder associated with immunosuppression | 4 (2.8) | 1 (1.0) | 1 (0.9) | 1. (0.0) |
| - Malignant disease | 1 (0.7) | 0 (0.0) | 0 (0.0) | 1. (0.0) |
| - Thyroid disease | 6 (4.2) | 0 (0.0) | 5 (4.6) | 1. (0.0) |
| **Medication (n (%))** |  |  |  |  |
| - Antiallergic agents | 8 (5.6) | 8 (8.3) | 6 (5.5) | 3 (5.1) |
| - Antidiabetic agents | 4 (2.8) | 0 (0.0) | 4 (3.7) | 0 (0.0) |
| - Angiotensin-converting enzyme inhibitor | 8 (5.6) | 0 (0.0) | 7 (6.4) | 0 (0.0) |
| - Angiotensin II Receptor Blocker | 7 (4.9) | 0 (0.0) | 5 (4.6) | 0 (0.0) |
| - Beta Blocker | 5 (3.5) | 0 (0.0) | 4 (3.7) | 0 (0.0) |
| - Inhaled Corticosteroids | 5 (3.5) | 1 (1.0) | 3 (2.8) | 0 (0.0) |
| - Immunosuppressive/   Immunomodulatory agents* | 3 (2.1) | 0 (0.0) | 2 (1.8) | 0 (0.0) |
| - Statins | 5 (3.5) | 0 (0.0) | 4 (3.7) | 0 (0.0) |
| - Vitamin D | 1 (0.7) | 2 (2.1) | 1 (0.9) | 0 (0.0) |
| **Vaccinated (%)** | 0 (0.0) | 0 (0.0) | 3 (2.8) | 0 (0.0) |
| **Number of families** | 66 | 54 |  |  |

See additional methods for definition of how samples were defined as being infected, seropositive, T cell positive, asymptomatic or symptomatic. Median time from positive PCR test to sample collection (adult n=70, children n=9 at T1, adult n=55, children n=7 at T2) and median time from symptoms onset to sample collection (adult n=62, children n=9 at T1, adult n=64, children n=8 at T2) are calculated using samples for which this data was available. Percentages of seropositive, T cell positive and PCR positive participants are calculated from infected participants. Disorders associated with immunosuppression included chronic rheumatic diseases, autoimmune disorders with known immunosuppressive medication, chronic inflammatory bowel disease. Immunosuppressive and immunomodulatory agents included systemic corticosteroids, monoclonal antibodies and cytostatic drugs. BMI Body Mass Index, IQR Interquartile Range, NA not applicable, PCR Polymerase Chain Reaction.

|  | T1 Cohort | |
| --- | --- | --- |
|  | Adult | Children |
| T-cell responders , n | 24 | 25 |
| T cell responders who tested positive in ≥1 of 4 commercial assays (n (%)) | 1 (4.2) | 2 (8.0) |
| T cell responders who tested negative in all commercial assays (n (%)) | 23 (95.8) | 23 (92.0) |

**Extended Data Table 2 – Assessment of negative serological status in T cell responders using commercial serology**

T-cell responders were defined as being tested positive for SI or SII or both within the ELISPOT assay but negative in MULTICOV-AB and with no reported positive PCR test. To confirm their negative serological status, the samples were also analysed with four commercially available assays (Roche ELECSYS N pan-Ig, EuroImmun S1 IgG and IgA, Siemens RBD IgG). Numbers and percentages (of total) of samples which were either positive in 1 or more of the commercial assays or negative in all commercial assays are provided.

**Extended Data Table 3 – Analysis of correlation between SI and SII magnitude with antibody titers**

| ADULT | SI | | | SII | | |
| --- | --- | --- | --- | --- | --- | --- |
|  | Pearson | R^2^ | ρ | Pearson | R^2^ | ρ |
| Spike | 0.11 | 0.01 | 0.29 | 0.05 | <0.01 | 0.63 |
| RBD | 0.08 | <0.01 | 0.43 | 0.06 | <0.01 | 0.53 |
| S1 | 0.16 | 0.03 | 0.11 | 0.13 | 0.02 | 0.21 |
| S2 | 0.44 | 0.19 | <0.001 | 0.33 | 0.05 | 0.001 |
| Nucleocapsid | 0.14 | 0.02 | 0.18 | 0.22 | 0.01 | 0.03 |
| CHILDREN | SI | | | SII | | |
|  | Pearson | R^2^ | ρ | Pearson | R^2^ | ρ |
| Spike | 0.41 | 0.17 | 0.03 | 0.12 | 0.01 | 0.54 |
| RBD | 0.22 | 0.05 | 0.26 | 0.01 | <0.01 | 0.95 |
| S1 | -0.12 | 0.01 | 0.55 | 0.21 | 0.04 | 0.28 |
| S2 | -0.03 | <0.01 | 0.87 | 0.27 | 0.07 | 0.17 |
| Nucleocapsid | -0.06 | <0.01 | 0.77 | 0.14 | 0.02 | 0.49 |

Linear regression with Pearson r used to determine strength of correlation, with Pearson r, r^2^ values and p-values provided.

**Extended Data Table 4 – statistical significance of correlations between SI/SII magnitude and individual symptoms**

SI

|  | Asymptomatic | Fever | Cough | Diarrhea | Dysgeusia | Flu | Cold |
| --- | --- | --- | --- | --- | --- | --- | --- |
| Asymptomatic |  |  |  |  |  |  |  |
| Fever | 8.10 x 10^-5^ |  |  |  |  |  |  |
| Cough | 9.50 x 10^-5^ | >0.99 |  |  |  |  |  |
| Diarrhea | 3.71 x 10^-3^ | >0.99 | >0.99 |  |  |  |  |
| Dysgeusia/Dysosmia | 1.65 x 10^-4^ | >0.99 | >0.99 | >0.99 |  |  |  |
| Influenza-like illness | 5.20 x 10^-5^ | >0.99 | >0.99 | >0.99 | >0.99 |  |  |
| Cold-like illness | >0.99 | >0.99 | >0.99 | >0.99 | >0.99 | >0.99 |  |

SII

|  | Asymptomatic | Fever | Cough | Diarrhea | Dysgeusia | Flu | Cold |
| --- | --- | --- | --- | --- | --- | --- | --- |
| Asymptomatic |  |  |  |  |  |  |  |
| Fever | 3.31 x 10^-4^ |  |  |  |  |  |  |
| Cough | 5.78 x 10^-4^ | >0.99 |  |  |  |  |  |
| Diarrhea | 8.36 x 10^-3^ | >0.99 | >0.99 |  |  |  |  |
| Dysgeusia/Dysosmia | 3.27 x 10^-4^ | >0.99 | >0.99 | >0.99 |  |  |  |
| Influenza-like illness | 2.15 x 10^-3^ | >0.99 | >0.99 | >0.99 | >0.99 |  |  |
| Cold-like illness | >0.99 | >0.99 | 0.96 | 0.53 | >0.99 | >0.99 |  |

Adjusted p-values as calculated by two-way ANOVA (Kruskal-Wallis) with Dunn’s multiple comparison test.

**Extended Data Table 5 – SARS-CoV-2 specific and cross-reactive HLA class I and HLA-DR restricted T cell epitope compositions.**

| SARS-CoV-2 specific HLA class I restricted EC (SI) | | |
| --- | --- | --- |
| Sequence | ORF | HLA |
| LTDEMIAQY | ORF2 spike | A*01 |
| ALSKGVHFV | ORF3 | A*02 |
| LLLLDRLNQL | ORF9 nucleocapsid | A*02 |
| QLRARSVSPK | ORF7 | A*03 |
| KTFPPTEPKK | ORF9 nucleocapsid | A*03 |
| ASMPTTIAK | ORF1 | A*11 |
| ATEGALNTPK | ORF9 nucleocapsid | A*11 |
| VYIGDPAQL | ORF1 | A*24 |
| VYFLQSINF | ORF3 | A*24 |
| FPRGQGVPI | ORF9 nucleocapsid | B*07 |
| NPANNAAIVL | ORF9 nucleocapsid | B*07 |
| FVKHKHAFL | ORF1 | B*08 |
| SELVIGAVIL | ORF5 membrane | B*40 |
| MEVTPSGTWL | ORF9 nucleocapsid | B*40 |
| YYQLYSTQL | ORF3 | C*07 |
| NRFLYIIKL | ORF5 membrane | C*07 |

| SARS-CoV-2 specific HLA-DR restricted EC (SII) | | |
| --- | --- | --- |
| Sequence | ORF | HLA |
| IGYYRRATRRIRGGD | ORF9 nucleocapsid | DR |
| AIVLQLPQGTTLPKG | ORF9 nucleocapsid | DR |
| YKHWPQIAQFAPSAS | ORF9 nucleocapsid | DR |
| LSYYKLGASQRVAGD | ORF5 membrane | DR |
| INVFAFPFTIYSLLL | ORF10 | DR |

| Coronavirus cross-reactive HLA class I restricted EC (CI) | | |
| --- | --- | --- |
| Sequence | ORF | HLA |
| TTDPSFLGRY | ORF1 | A*01 |
| RTFKVSIWNLDY | ORF6 | A*01 |
| KLFAAETLK | ORF1 | A*03 |
| QYIKWPWYI | ORF2 spike | A*24 |
| TPKYKFVRI | ORF1 | B*08 |
| DLKGKYVQI | ORF1 | B*08 |
| EAFEKMVSL | ORF1 | B*08 |
| YEGNSPFHPL | ORF7 | B*40 |
| IEYPIIGDEL | ORF1 | B*40 |

| Coronavirus cross-reactive HLA-DR restricted EC (CII) | | |
| --- | --- | --- |
| Sequence | ORF | HLA |
| KDGIIWVATEGALNT | ORF9 nucleocapsid | DR |
| GTWLTYTGAIKLDDK | ORF9 nucleocapsid | DR |
| RWYFYYLGTGPEAGL | ORF9 nucleocapsid | DR |
| ASWFTALTQHGKEDL | ORF9 nucleocapsid | DR |
| ASAFFGMSRIGMEVT | ORF9 nucleocapsid | DR |
| LLLLDRLNQLESKMS | ORF9 nucleocapsid | DR |
| FYVYSRVKNLNSSRV | ORF4 envelope | DR |
| IWNLDYIINLIIKNL | ORF6 | DR |
| QEEVQELYSPIFLIV | ORF7 | DR |
| SKWYIRVGARKSAPL | ORF8 | DR |

EC: epitope composition, orf: open reading frame, SI: specific class I, SII: specific class II, CI: cross-reactive class I, CII: cross-reactive class II. The peptides used in this manuscript were previously published as part of Nelde A, et al. SARS-CoV-2-derived peptides define heterologous COVID-19-induced T cell recognition. Nature Immunology 22, 74-85 (2021).

**Extended Data Table 6 – Antigens included within MULTICOV-AB**

| Disease | Antigen | Manufacturer | Cat. No |
| --- | --- | --- | --- |
| SARS-CoV-2 | Spike Trimer | NMI | - |
| SARS-CoV-2 | RBD | NMI | - |
| SARS-CoV-2 | S1 domain | NMI | - |
| SARS-CoV-2 | S2 domain | Sino Biological | 40590 |
| SARS-CoV-2 | Nucleocapsid | Aalto Bioreagents | 6404-b |
| hCoV-OC43 | S1 domain | NMI | - |
| hCoV-OC43 | Nucleocapsid | NMI | - |
| hCoV-NL63 | S1 domain | NMI | - |
| hCoV-NL63 | Nucleocapsid | NMI | - |
| hCoV-HKU1 | S1 domain | NMI | - |
| hCoV-HKU1 | Nucleocapsid | NMI | - |
| hCoV-229E | S1 domain | NMI | - |
| hCoV-229E | Nucleocapsid | NMI | - |

List of antigens included in MULTICOV-AB in this study, including information about their manufacturer, and if available, their category number. Full information on the NMI produced antigens can be found at Becker M, et al. Immune response to SARS-CoV-2 variants of concern in vaccinated individuals. Nat Commun 12, 3109 (2021).

**Extended Data Figure 1 – Flowchart of study cohort**


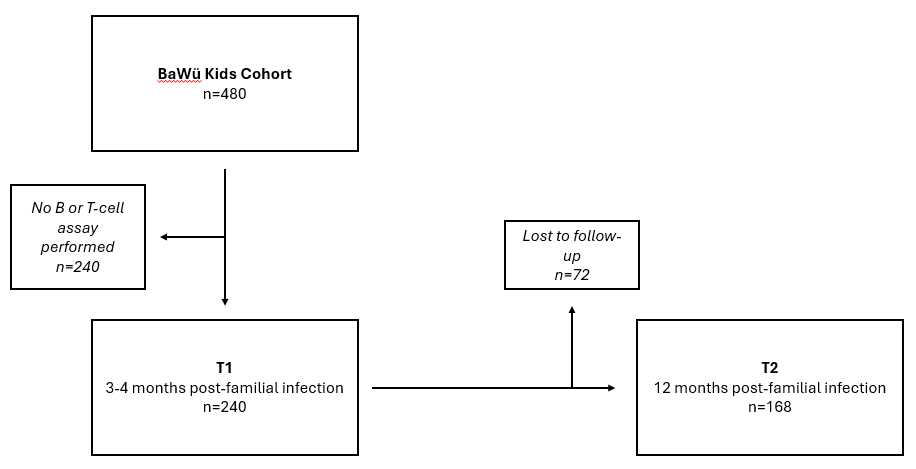


Flowchart detailing how samples were included/excluded for use in this specific study. Samples were collected at 2 timepoints: 3-4 months post-familial infection (T1 cohort) and 12 months post-familial infection (T2 cohort). For inclusion in T1, all participants had to have their humoral and cellular immune responses analysed using both the MULTICOV-Ab and IFN-γ ELISPOT assay. Between T1 and T2, 72 samples were lost to follow up.

**Extended Data Figure 2 – Correlation of T cell magnitude with antibody titer**

While T cell magnitude did correlate with antibody status (see **Figure 2**), it is not associated with any B cells against any particular antigen. Dot plots showing correlation between SI (a, c, e and g) and SII (b, d, f and h) magnitude against Spike, (a and b), RBD (c and d), S1 domain (e and f) and S2 domain (g and h) titers. Correlation analysis (linear regression with Pearson correlation) for each plot is included as **Extended Data Table 3**. Adults – blue, Children – orange.

**Extended Data Figure 3 – Exemplary ELISPOT assays of SARS-CoV-2 antibody negative and T cell positive individuals at T1**

**
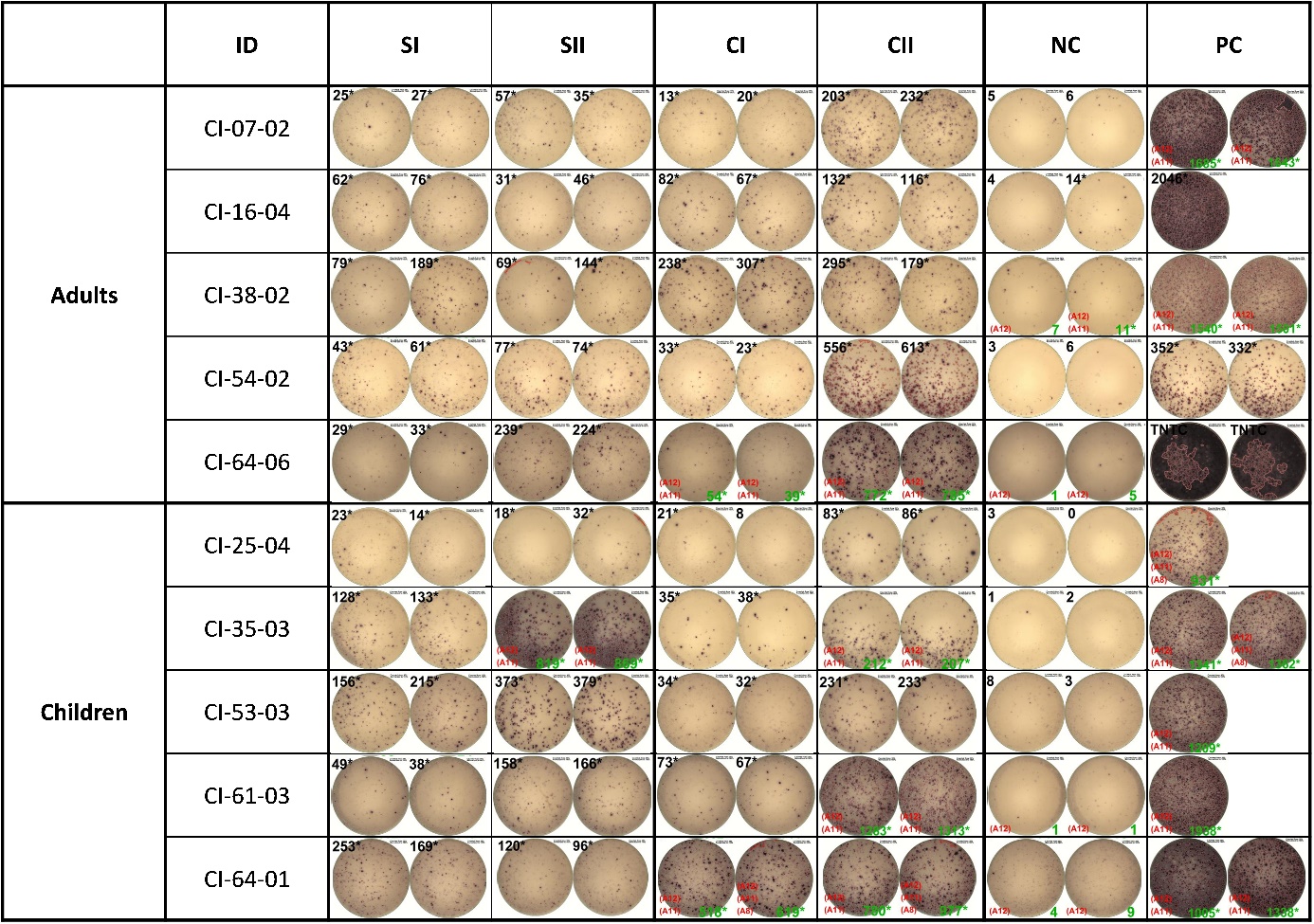
**

Exemplary images of IFN-γ ELISPOT assays of n=10 individuals (5 adults and 5 children) who were B cell negative and T cell positive at T1. Representative replicates are shown. ID = study individual identifier; SI = specific HLA class I EC; SII = specific HLA-DR EC; CI = cross-reactive HLA class I EC; CII = cross-reactive HLA-DR EC; NC = negative control; PC = positive control. Spot counts are depicted in black at the upper left corner or in green at the lower right corner of each well, respectively.

**Extended Data Figure 4 – ELISPOT assay performance comparison between PCR-confirmed previously infected samples and pre-pandemic samples**

Samples from individuals with PCR-confirmed infections (n=79) and pre-pandemic individuals (n=31) were compared to confirm assay performance and specificity of the SARS-CoV-2 specific peptide pools used (ECs SI and SII). Overall, 73 of 79 samples were classified as positive using the ELISPOT (61 SI and SII positive, 2 SI positive only, 10 SII positive only). 0 of 31 pre-pandemic samples were classified as positive. Both SI magnitude (a) and SII magnitude (b) are shown. Scattered dot plots with the line representing the mean. 0 values were manually adjusted to 0.01 for display purposes only. The dashed line represents the raw CO value used for positivity. Statistical analysis was Mann-Whitney U with **** indicating a p<0.0001.
